# Estimating the Global Prevalence of Inadequate Zinc Intake from National Food Balance Sheets: Effects of Methodological Assumptions

**DOI:** 10.1371/journal.pone.0050565

**Published:** 2012-11-29

**Authors:** K. Ryan Wessells, Gitanjali M. Singh, Kenneth H. Brown

**Affiliations:** 1 Department of Nutrition, University of California Davis, Davis, California, United States of America; 2 Department of Nutrition, Harvard School of Public Health, Boston, Massachusetts, United States of America; Aga Khan University, Pakistan

## Abstract

**Background:**

The prevalence of inadequate zinc intake in a population can be estimated by comparing the zinc content of the food supply with the population’s theoretical requirement for zinc. However, assumptions regarding the nutrient composition of foods, zinc requirements, and zinc absorption may affect prevalence estimates. These analyses were conducted to: (1) evaluate the effect of varying methodological assumptions on country-specific estimates of the prevalence of dietary zinc inadequacy and (2) generate a model considered to provide the best estimates.

**Methodology and Principal Findings:**

National food balance data were obtained from the Food and Agriculture Organization of the United Nations. Zinc and phytate contents of these foods were estimated from three nutrient composition databases. Zinc absorption was predicted using a mathematical model (Miller equation). Theoretical mean daily per capita physiological and dietary requirements for zinc were calculated using recommendations from the Food and Nutrition Board of the Institute of Medicine and the International Zinc Nutrition Consultative Group. The estimated global prevalence of inadequate zinc intake varied between 12–66%, depending on which methodological assumptions were applied. However, country-specific rank order of the estimated prevalence of inadequate intake was conserved across all models (*r* = 0.57–0.99, *P*<0.01). A “best-estimate” model, comprised of zinc and phytate data from a composite nutrient database and IZiNCG physiological requirements for absorbed zinc, estimated the global prevalence of inadequate zinc intake to be 17.3%.

**Conclusions and Significance:**

Given the multiple sources of uncertainty in this method, caution must be taken in the interpretation of the estimated prevalence figures. However, the results of all models indicate that inadequate zinc intake may be fairly common globally. Inferences regarding the relative likelihood of zinc deficiency as a public health problem in different countries can be drawn based on the country-specific rank order of estimated prevalence of inadequate zinc intake.

## Introduction

Adequate zinc nutrition is essential for adequate growth, immunocompetence and neurobehavioral development of young children and normal pregnancy outcomes [1]. The World Health Organization (WHO), UNICEF, the International Atomic Energy Agency (IAEA) and the International Zinc Nutrition Consultative Group (IZiNCG) recommend that plasma zinc concentration, dietary zinc intake and prevalence of stunting are the best indicators of population risk of zinc deficiency [2]. Although few countries have collected relevant data from a representative sample of the national population, existing estimates suggest that zinc deficiency may be fairly common [3]–[7]. In lieu of such information, national food balance sheets, which are produced annually by the Food and Agriculture Organization (FAO) of the United Nations, can be used to estimate the quantities of total and absorbable zinc in national food supplies. When compared to the respective population’s theoretical requirements for zinc, the results may provide useful information regarding the estimated prevalence of inadequate zinc intake in the population.

Two previous analyses, conducted in 2001 and 2005, have used this ecological approach, estimating the global prevalence of inadequate zinc intake at 48.9% and 20.5%, respectively. The difference between estimates was due primarily to changes in methodological assumptions regarding food processing methods and updated information regarding zinc requirements and the fractional absorption of dietary zinc [8], [9].

The objectives of the current analysis were to (1) update the country- and region-specific estimated prevalence of dietary zinc inadequacy, focusing on four major sources of uncertainty in this analysis and evaluating the effects of different assumptions on estimates of the prevalence of inadequate zinc intake, and (2) generate a model considered to provide the best estimate of the prevalence of inadequate zinc intake, given the current state of knowledge. The different input parameters for the model are as follows:

1. Alternative global and regional nutrient composition databases to estimate the amount of zinc and phytate in the national food supplies, recognizing that reported zinc and phytate values of foods may vary substantially by agricultural conditions, plant cultivar and methods of laboratory analysis.
2. Different age- and sex-specific estimates of average physiological requirements for absorbed zinc, as well as requirements for total dietary zinc intake, as developed by the Food and Nutrition Board of the Institute of Medicine (FNB/IOM) and the International Zinc Nutrition Consultative Group (IZiNCG). Both sets of estimates were used because of a lack of consensus regarding zinc requirements.
3. Different methods to estimate the fractional absorption of zinc (FAZ), using either mathematical modeling, based on zinc and phytate contents of the food supply, or P:Zn molar ratio cut-offs.
4. Alternative assumptions regarding inter-individual variation in dietary zinc intake, ranging from 20–30%, due to limited data from nationally representative surveys in populations of interest.

## Methods

The following steps were completed to estimate the prevalence of inadequate zinc intake on a country-specific basis, for each country with available data:

1. Calculation of country-specific data on the average daily per capita availability of major food commodities;
2. Calculation of the zinc and phytate contents of each food commodity;
3. Estimation of the absorbable zinc content of the daily food supply on a per country basis;
4. Calculation of the theoretical mean daily per capita physiological and dietary requirements for zinc, based on the age and sex distribution of the national population;
5. Comparison of the absorbable or total dietary zinc content of the food supply with the population’s theoretical mean physiological requirement or dietary requirement for zinc, respectively; and
6. Estimation of the prevalence of inadequate zinc intake in a population and calculation of country-specific rank order by estimated prevalence.

Each of these steps is further explained in the following paragraphs.

### Calculation of Country-specific Data on the Average Daily per Capita Availability of Major Food Commodities

The FAO publishes annual food balance sheets (FBS), which provide country-specific data on the quantities of foods available for human consumption. To smooth differences in within-country inter-year variability, the present analyses are based on the mean availability of commodity foods from 2003 to 2007, encapsulating the latest five years of data currently available in the public domain. Data were downloaded for 210 countries and territories with available information for the period from 1998–2007 [10]. All FAO FBS variables were examined to identify outliers, defined as 3 SD above or below the country mean for that variable and/or a 3-fold increase or decrease between two-consecutive years; approximately 3% of the data were identified as outliers. Each outlier was visually inspected and assigned an outlier pattern (96% were a sudden spike or dip and 2% were a sudden discontinuity; the remainder were either part of a larger trend, or occurred in 2007, so that a pattern could not be assigned). Outliers explained by a sudden spike or dip were assigned the average of the three previous years and up to three subsequent years. Outliers resulting from a sudden discontinuity in the data were selectively corrected when the discontinuity appeared to be the result of a change in reporting, as opposed to an actual change in the availability of the food commodity; values were adjusted by the amount of the discontinuity to allow for a continuous trend. When country-specific information was missing (e.g. Afghanistan, post-Soviet republics), data for each food commodity was imputed using the Imputed Chain Equation (ICE) module (STATA 10), with data year, five-year lags and leads, and the regional average as covariates. In instances when countries dissolved during the period from 1998–2007 (e.g. Serbia and Montenegro), countries resulting from the split were assigned the values of the parent country for the period prior to the split.

### Calculation of the Zinc and Phytate Contents of Each Food Commodity

The FAO FBS report 95 ‘standardized’ food commodities, the majority of which are created using one of two methods. The first standardization method aggregates similar foods into one commodity (e.g. ‘poultry meat’, ‘beans’). The second standardization method reverts processed foods back to the original commodity (i.e. fresh, evaporated and dry milks, yogurt and cheeses are all reported as ‘milk-excluding butter’). The FAO provides definitions and a listing of possible foods included in each standardized commodity [11]. Data on standardized food commodities available for human consumption are expressed in terms of quantity (kg per capita per year), as well as in terms of dietary energy (kcal per capita per day), and were obtained by applying FAO nutrient composition data to all primary and processed products [12]. However, neither the proportion contributed by individual foods, nor the type or extent of processing applied to the primary product are reported on the national food balance sheets. Therefore, all analyses were calculated based on the daily per capita caloric availability of the food commodities (i.e. mg Zn/100 kcal) rather than on a weight basis, to more accurately estimate the amount available for consumption.

As mentioned above, the first standardization method aggregates similar foods into one standardized food commodity. A previous review of the zinc and phytate contents of the individual foods included in each of the FAO standardized food commodities created by this standardization method concluded that there was limited intra-category variability in the amounts of these two food components [8]. Therefore, in the present analysis, the zinc and phytate contents of FAO standardized food commodities based on aggregation were calculated as the mean of these values for all appropriate foods defined for each food commodity for which there were nutrient composition data available [11]; all foods were assumed to contribute equal weights. For example, the zinc content of ‘poultry meat’ was calculated as the average zinc content of chicken, goose/guinea fowl, turkey and duck meats. For fish and other standardized aquatic food commodities created by aggregation, mean zinc and phytate levels were calculated based on International Standard Statistical Classification of Aquatic Animals and Plants (ISSCAAP) Divisions/Groups and the predominant genera or species in each category, assuming each individual item contributed an equal weight to the standardized commodity [13]. The nutrient values for oysters and for molasses and maple syrup (included in the definition of the commodities ‘crustaceans’ and ‘sweeteners’, respectively), were not included in the present analyses, as the zinc content of these foods is very high relative to other foods in their respective standardized commodities, and they are likely to be available in relatively small amounts compared with other foods in the same categories. A detailed listing of individual foods aggregated into each standardized food commodity and their relative weighting factors is available as online supporting material **(Table S1)**.

The second standardization method reverts processed foods back to the original primary commodity. For FAO standardized food commodities created according to this standardization method, zinc and phytate values were assumed to be those of either the representative whole-grain non-fortified flour for cereals or the non-processed primary food for all other commodities (e.g. ‘fresh milk, whole’ nutrient values applied for ‘milk-excluding butter’ commodity).

In the previous analysis conducted by this group, zinc and phytate contents of each food commodity were obtained from the WorldFood System International Mini-list (IML) [14]. For the current analysis, we also calculated the zinc and phytate contents of each food commodity using the Nutrition Data System for Research (NDSR, Nutrition Coordinating Center, University of Minnesota) [15]. Inter-database variations in the calculated zinc and phytate contents of the aggregated food commodities (mg/100 g) were then compared. It was decided *a priori* that when the percent difference in either zinc or phytate content for any aggregated food commodity was greater than 25% between databases, or the amount of zinc or phytate from a single aggregated food commodity accounted for greater than 5% of the daily availability of that food component for human consumption in any region, attempts would be made to resolve the differences between databases and create best estimates. However, 80% of the food commodities which contained either zinc or phytate met the aforementioned criteria; therefore all food commodities were re-evaluated. KRW reviewed the available reference data underlying the nutrient values for the NDSR and IML databases. Data for the new composite database were obtained from the two aforementioned databases, the USDA Nutrient Database for Standard Reference, Release 23 (USDA SR23) [16], the INFOODS Regional Nutrient Database for West Africa [17], *Food Phytates,* edited by Reddy *et al.* [18], and current literature indexed in ScienceDirect, Agricola and PubMed. We have thus created a new composite database with zinc and phytate values for the food commodities, which serves as the “best-estimate” food composition database for subsequent analyses. Following procedures similar to those used in the development of the International Mini-list [14], Zn values were obtained from the USDA SR23 and phytate values were estimated as the midpoint of the range reported by Reddy *et al*., when available; all values were checked for internal and external consistency. The zinc and phytate values for each of the aggregated food commodities, as well as documentation of data sources, are available as online supporting material for each database **(Table S1)**. The subsequent analyses were conducted using each of the three nutrient databases (IML, NDSR and our composite database) to examine the effects of different estimates of zinc and phytate contents of the food commodities on the estimated prevalence of inadequate zinc intake.

Whole-grain cereals typically contain more zinc and phytate than decorticated grains and low extraction (i.e. refined) flours, due to higher concentrations of zinc and phytate in the bran and germ as compared to the endosperm. In addition, food processing techniques, such as soaking, fermentation and nixtamalization, can reduce the phytate contents of cereals and legumes, thus affecting the bioavailability of zinc in the processed foods. We attempted to locate country- and/or region-specific data on the methods and rates of extraction and processing of cereals and legumes, as well as the effects of extraction and processing on the zinc and phytate contents of the food commodities. However, such information is lacking and may be highly variable within and between countries. To re-evaluate and improve previously developed regional estimates, we solicited information from international research centers, experts in the field, and current literature searchable through ScienceDirect, Agricola and PubMed. The regional processing and extraction assumptions that were ultimately used for each region, and the effects of extraction and processing on zinc and phytate contents of the aggregated food commodities, are provided as online supporting material **(Table S2, Table S3)**. Due to the relatively limited number of countries implementing mandatory national zinc fortification programs prior to 2007, no assumptions were made regarding the zinc fortification of flour [19].

### Estimation of the Absorbable Zinc Content of the Daily Food Supply on a per Country Basis

The proportion of dietary zinc that is absorbed is determined primarily by the total zinc and phytate content of the diet. The fractional absorption of zinc (FAZ, %) decreases with increasing zinc intake, although the total absorption of zinc (TAZ, mg) increases. Phytate, a phosphorus storage molecule in plants, is a strong chelator of zinc; the phytate: zinc complex passes through the intestinal tract unabsorbed. The inhibitory effect of phytate on zinc absorption is dose-dependent [1]. The Miller equation, which is a saturation response model of zinc absorption as a function of dietary zinc and phytate [20], [21], was used to predict the FAZ and the absorbable zinc content of the daily food supply on a per country basis. Predictions generated based on this equation are considered to be the “best-estimate” estimates for subsequent analyses.

### Calculation of the Theoretical Mean Daily per Capita Requirement for Zinc, Based on the Age and Sex Distribution of the National Population

Theoretical mean daily per capita requirements for zinc can be calculated based on either estimated average physiological requirements for absorbed zinc or estimated average dietary requirements for total zinc intake. These latter requirements are termed the “estimated average requirements” (EAR) by the Dietary Reference Intakes (DRI) of the FNB/IOM; however, for the purpose of this analysis they will be referred to as “dietary requirements”. Due to a lack of consensus regarding zinc requirements, we conducted separate analyses using both the physiological and dietary requirements.

### Physiological Requirement for Absorbed Zinc

The FNB/IOM, WHO/FAO/IAEA, and IZiNCG have each proposed different age- and sex-specific physiological requirements for absorbed zinc. Although all methods use the same general factorial approach, estimated requirements reflect different criteria for inclusion of studies in the analyses (age, sex, nationality and diet-type), different reference body weights, and different statistical methods with or without weighting studies by sample size [1]. The IZiNCG physiological requirements are considered to be the “best-estimate” requirements for zinc in these analyses, as they are intended to be generalizable internationally. However, given the current lack of consensus, we used both FNB/IOM and IZiNCG values to calculate two different estimates of the theoretical mean daily per capita physiological requirement for absorbed zinc **(** **Table 1** **)** [1], [22]. To do so, the estimated average physiological requirements for zinc in each age and sex grouping were weighted according to the mean national population distributions over the five-year period of interest from 2003–2007. Population data were obtained from the Institute for Health Metrics and Evaluation (IHME, University of Washington) and based on the 2010 Revision of the World Population Prospects, available from the Population Division of the United Nations Department of Economic and Social Affairs [23]. Population data were not available for 22 territories and states, mostly small island entities, for which there were food balance sheet data; these states were excluded from further analyses. Children less than six months of age were assumed to be exclusively breastfed, and were not included in the estimated mean physiological requirements. However, increased physiological requirements of pregnant and lactating (<6 mo post-partum) women were included in the estimated means. The number of pregnant women was calculated by multiplying the total number of children less than one year of age * 0.729; the number of lactating women was assumed to be equal to the number of children <6 months, recognizing that this underestimates the number of women breastfeeding in many countries. However, the concentration of zinc in breast milk declines rapidly from birth to 6 months post-partum [24]. In addition, the amount of milk transferred to breastfed infants decreases with the introduction of complementary foods [25]. Thus, we assumed that maternal zinc requirements declined once infants reached 6 months of age. The subsequent analyses were conducted using both the FNB/IOM and IZiNCG estimated population mean physiological requirements, in conjunction with the composite nutrient database and the absorbable zinc content of the national food supply estimated by the Miller equation, to investigate the effects of differing estimated physiological requirements on the estimated prevalence of inadequate zinc intake.

**Table 1:** Estimated physiologic requirements and dietary requirements for zinc (mg/d), by population group, as developed by the FNB/IOM and IZiNCG^1^.

	Physiological Requirements		Dietary Requirements		
Population Group	FNB/IOM	IZiNCG	FNB/IOM	IZiNCG P:Zn≤18	IZiNCG P:Zn >18
6–11 mo	0.84	0.84	2.5	3	4
1–3 y	0.74	0.53	2.5	2	2
4–8 y	1.20	0.83	4	3	4
9–13 y	2.12	1.53	7	5	7
14–18 y, M	3.37	2.52	8.5	8	11
14–18 y, F	3.02	1.98	7.3	7	9
≥19 y, M	3.84	2.69	9.4	10	15
≥19 y, F	3.30	1.86	6.8	6	7
Additional requirement for pregnancy	0.39	0.70	2.7	2	3
Additional requirement for lactation	1.35	1.00	3.6	1	1

1 FNB/IOM, Food and Nutrition Board of the Institute of Medicine; IZiNCG, International Zinc Nutrition Consultative Group, P:Zn, phytate: zinc molar ratio.

### Dietary Requirements for Total Zinc Intake

As an alternative strategy, given the aforementioned uncertainties surrounding the phytate contents of food commodities, and lack of consensus regarding physiological requirements, we also used age- and sex-specific estimated average dietary requirements for total zinc intake put forth by the FNB/IOM [22], as well as age-, sex- and diet-type-specific requirements developed by IZiNCG [1], to calculate two different estimates of the theoretical mean daily per capita dietary requirement for total zinc intake **(** **Table 1** **)**. The IZiNCG dietary requirements account for the effects of phytate on total zinc intake requirements, whereas the FNB/IOM dietary requirements do not consider the effects of phytate. To categorize countries according to diet-type, so as to apply the appropriate dietary requirement, as developed by IZiNCG, we first calculated phytate: zinc (P:Zn) molar ratios to estimate zinc absorption categorically (mixed or refined-vegetarian diets, high absorption, P:Zn≤18; unrefined, cereal based diets, low absorption, P:Zn >18). The theoretical mean daily per capita dietary requirement was calculated using the aforementioned weighting method based on the age and sex distribution of the population. The subsequent analyses were conducted using both the FNB/IOM and IZiNCG population mean theoretical dietary requirements, in conjunction with the composite nutrient database and availability of zinc in the food supply (mg/capita/d), to investigate the effects of differing dietary requirements on the estimated prevalence of inadequate zinc intake.

### Comparison of the Zinc Content of the Food Supply with the Population’s Theoretical Mean Requirement

To calculate the percentage of the mean physiological requirement for zinc that is available in the national food supply, we divided the estimated absorbable zinc content of the national food supply by the theoretical mean national requirement for absorbed zinc. We also divided the estimated daily per capita availability of zinc (mg/d) by the theoretical mean national requirement for dietary zinc intake to calculate the percentage of the mean dietary requirement for zinc that is available in the food supply. We obtained six estimates of the percentage of the mean requirement for zinc that is available in the national food supply, using different combinations of methodological assumptions, as indicated in **Table 2**.

**Table 2:** Combinations of methodological assumptions used to estimate the percentage of the theoretical requirement for zinc available in the national food supply^1^.

Nutrient Database	Zn requirements	Zn availability in food supply
Composite	IZiNCG Physiological	Absorbable zinc (Miller equation)
IML	IZiNCG Physiological	Absorbable zinc (Miller equation)
NDSR	IZiNCG Physiological	Absorbable zinc (Miller equation)
Composite	FNB/IOM Physiological	Absorbable zinc (Miller equation)
Composite	IZiNCG Dietary	Total dietary zinc
Composite	FNB/IOM Dietary	Total dietary zinc

1 IML, WorldFood System International Mini-list; NDSR, Nutrition Data System for Research; IZiNCG, International Zinc Nutrition Consultative Group; FNB/IOM, Food and Nutrition Board of the Institute of Medicine.

### Estimation of the Prevalence of Inadequate Zinc Intake in a Population and Calculation of Country-specific Rank Order by Estimated Prevalence

We then applied a method akin to the Estimated Average Requirement (EAR) cut-point method described by the IOM [26] to estimate the prevalence of inadequate zinc intake in a population, using each of the six aforementioned calculations. The EAR cut-point method provides a good estimate of the prevalence of inadequate intakes in a population; under most circumstances the proportion of a population that has usual absorbable zinc intakes less than the mean physiological requirement (or usual dietary intakes less than the mean dietary requirement) is approximately the same as the proportion of the population with intakes below their actual requirements. This method assumes that the distributions of requirement and intake are independent, the requirement distribution is symmetrical around the mean physiological or dietary requirement, the variability of intakes is greater than the variability of requirements and the true prevalence of inadequate zinc intake is between ∼8–92% [26]. In the present analysis, we assumed a 25% inter-individual coefficient of variation (CV) in zinc intake, based on existing data sets for which intake distribution, corrected for intra-individual intake variability, has been determined [27]–[31]. This method was applied to the estimates of the percentage of the mean physiological, or dietary, requirements for zinc that are available in the national food supply. Given the limited data available on inter-individual variation intake, we also estimated the effects of assuming inter-individual CVs of 20% or 30% on the estimated prevalence of inadequate zinc intake, holding the other assumptions constant (i.e., by using the composite nutrient database, IZiNCG physiological requirements and the Miller equation).

Due to the numerous methodological assumptions inherent in this analysis, and the lack of consensus regarding zinc requirements and nutrient composition of food commodities, we also ranked countries by estimated prevalence of inadequate zinc intake and examined the conservation of rank among models based on different assumptions.

### Generation of a “Best-estimate” Model

As noted previously, we attempted to identify the most appropriate assumption at each step in the methodological process, given the current state of knowledge regarding nutrient composition of foods, zinc requirements, the effects of zinc and phytate intakes on zinc absorption, and inter-individual variability in intake. We then used the totality of these assumptions to generate a “best-estimate” model to determine the prevalence of inadequate zinc intake, which is comprised of zinc and phytate data from the composite nutrient database, IZiNCG physiological requirements for absorbed zinc, the Miller equation to estimate the fractional absorption of zinc and a 25% CV in inter-individual variation in intakes.

### Statistical Analyses

Regional classifications are based on the reporting regions of the Global Burden of Diseases, Injuries, and Risk Factors 2010 Study, and are grouped according to geographical location and dietary patterns **(Table S4)** [32]. Regional and global data were weighted by national population sizes. Bivariate associations between estimates of the prevalence of inadequate zinc intake generated by varying methodological assumptions, as well as the conservation of country-specific rank order by estimated prevalence of inadequate zinc intake among models, were assessed with Spearman correlations. All statistical analyses were completed using SAS System for Windows release 9.3 (SAS Institute, Cary, North Carolina). Data are presented as means±SD, unless otherwise noted. A *P* value <0.05 was considered statistically significant.

## Results

Regional data, based on the “best-estimate” model for the estimated prevalence of inadequate zinc intake in the population are presented in **Table 3**. The data are presented first for high-income countries, and then for the remaining regions in ascending order according to the estimated prevalence of inadequate zinc intake in the population. Based on this model, an estimated 17.3% of the global population has inadequate zinc intake, weighted by national population size. The regional prevalence of inadequate intake ranged from 6–7% in high-income regions and Southern and Tropical Latin America to 30% in South Asia.

**Table 3:** Regional means (± SD) for national data on the estimated prevalence of inadequate zinc intake for 188 countries, as calculated by varying assumptions to generate six different models^1^.

VARIABLE	High-income	Southern and TropicalL. America	China	Central and EasternEurope	Central and AndeanL. Americaand Carib.	Central Asia, North Africa and MiddleEast	East and SoutheastAsia and Pacific	Sub-SaharanAfrica	South Asia	Global
Number of Countries	30	5	3	20	27	28	21	48	6	188
Population (millions)	937.2	249.4	1337.7	330.1	301.4	481.4	606.8	757.8	1495.6	6497.5
Composite-IZiNCG PhysReq-Miller Equation	7.5±4.1	6.4±1.8	7.8±2.1	9.6±2.4	17±5.9	17.1±5.4	22.1±10.0	25.6±12.2	29.6±3.6	17.3±11.1
IML-IZiNCG Phys Req -Miller Equation	8.4±4.5	10.4±3.4	10.9±2.8	11.6±2.6	19±5.8	20.6±7.0	32.1±11.3	34.5±6.0	26.7±13.3	20.9±12.7
NDSR- IZiNCG Phys Req -Miller Equation	6.8±2.7	6.1±1.9	4.3±1.1	11.1±3.7	13.8±4.2	18.9±7.0	11.4±5.2	21.6±3.2	27.3±17.2	13.8±10.5
Composite-FNB/IOMPhys Req-Miller Equation	45.2±15.1	40.8±10.2	47.5±5.4	56.6±7.3	70.7±13.1	71.3±10.4	76.9±15.7	88.6±4.3	79.1±14.2	66.1±20.7
Composite-IZiNCGDietary Req	4.2±2.7	3.9±1.1	2.9±1.6	7.1±4.3	6.9±6.8	18.6±15.5	40.2±31.4	37.9±12	45.1±26.9	20.8±23.2
Composite-FNB/IOMDietary Req	5.1±3.2	5.2±1.5	3.7±1.9	8.7±5.1	4.8±5.6	18.4±17.5	25.4±12	14.1±6.8	26.7±22.8	12.1±13.3

1 Assumptions included nutrient composition database (Composite, IML or NDSR), Zn requirements (IZiNCG or FNB/IOM physiological requirements with the Miller equation to estimate zinc absorption and IZiNCG or FNB/IOM dietary requirements). All models assume a 25% inter-individual variation in zinc intake. Regional data are presented first for high-income countries, and then in ascending order (from left to right) according to the estimated prevalence of inadequate zinc intake. Regional classifications are based on the reporting regions of the Global Burden of Diseases, Injuries, and Risk Factors 2010 Study, and are grouped according to geographical location and dietary patterns (Table S3). Data are weighted by national population sizes. IZiNCG, International Zinc Nutrition Consultative Group; IML, WorldFood System International Mini-list; NDSR, Nutrition Data System for Research; FNB/IOM, Food and Nutrition Board of the Institute of Medicine; Phys Req, physiological zinc requirements; Dietary Req, dietary zinc requirements.

### Effect of Varying the Nutrient Composition Database on the Estimated Prevalence of Inadequate Zinc Intake

**Figure 1:**
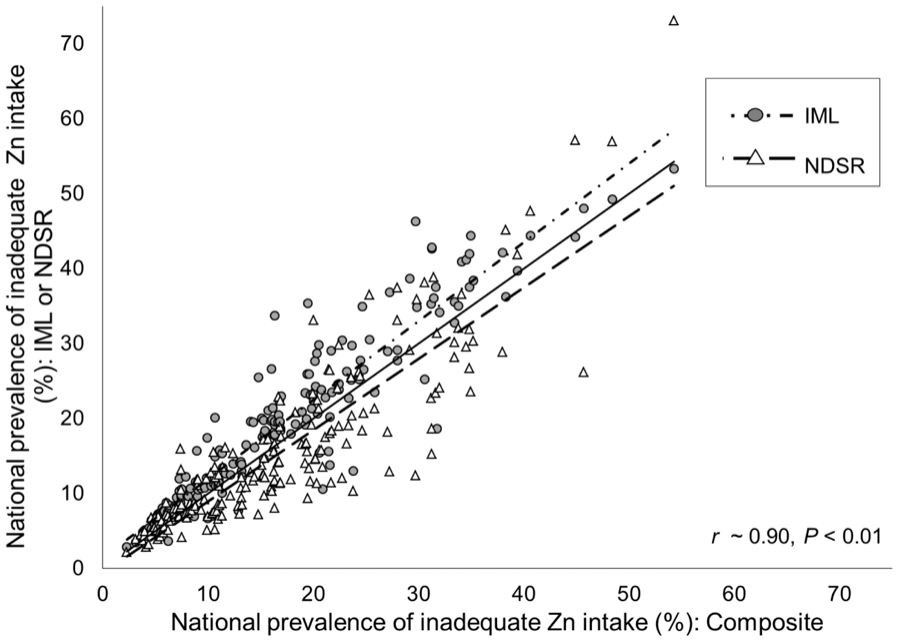
Associations between the national prevalence of inadequate zinc intake as estimated by different nutrient composition databases. Estimates are based on the Composite, WorldFood System International Mini-list (IML) and Nutrition Data System for Research (NDSR) nutrient composition databases. Physiological requirements developed by the International Zinc Nutrition Consultative Group (IZiNCG), the Miller equation for absorbed zinc and a 25% inter-individual CV in intake are constant across estimates. The dashed lines represent the linear regression lines and the solid line represents the line of identity (intercept = 0, slope = 1). N = 188 countries.

Holding assumptions regarding physiological zinc requirements (IZiNCG) and absorbed zinc (Miller equation) constant, the IML and NDSR databases provide estimates of the global prevalence of inadequate zinc intake of 20.9% and 13.8%, respectively, compared with the 17.3% estimated by the composite database **(** **Table 3** **;** **Figure 1**). The major reasons for these differences were the lower estimates of total zinc, phytate, and P:Zn molar ratios in national food supplies, and resultant higher estimates of FAZ and total absorbable zinc in the global food supply calculated using the NDSR database as compared to the IML. Applying the composite database resulted in intermediate estimates (data not shown). Country-specific percentages of energy available from cereals, particularly rice, were correlated with the inter-database difference in estimated prevalence of inadequate zinc intake (Composite vs. IML, r = −0.31; Composite vs. NDSR, r = 0.69; *P*<0.001), due to dissimilarities in the reported zinc and phytate contents, and P:Zn molar ratio, of rice among databases. However, although the estimated prevalence of inadequate zinc intake varied, country-specific rank order of estimated prevalence of inadequate intake was highly conserved when different databases were used to estimate zinc and phytate contents **(**r ∼ 0.90, *P*<0.001**)**.

### Effect of Different Estimates of Physiological Requirements on the Estimated Prevalence of Inadequate Zinc Intake

**Figure 2:**
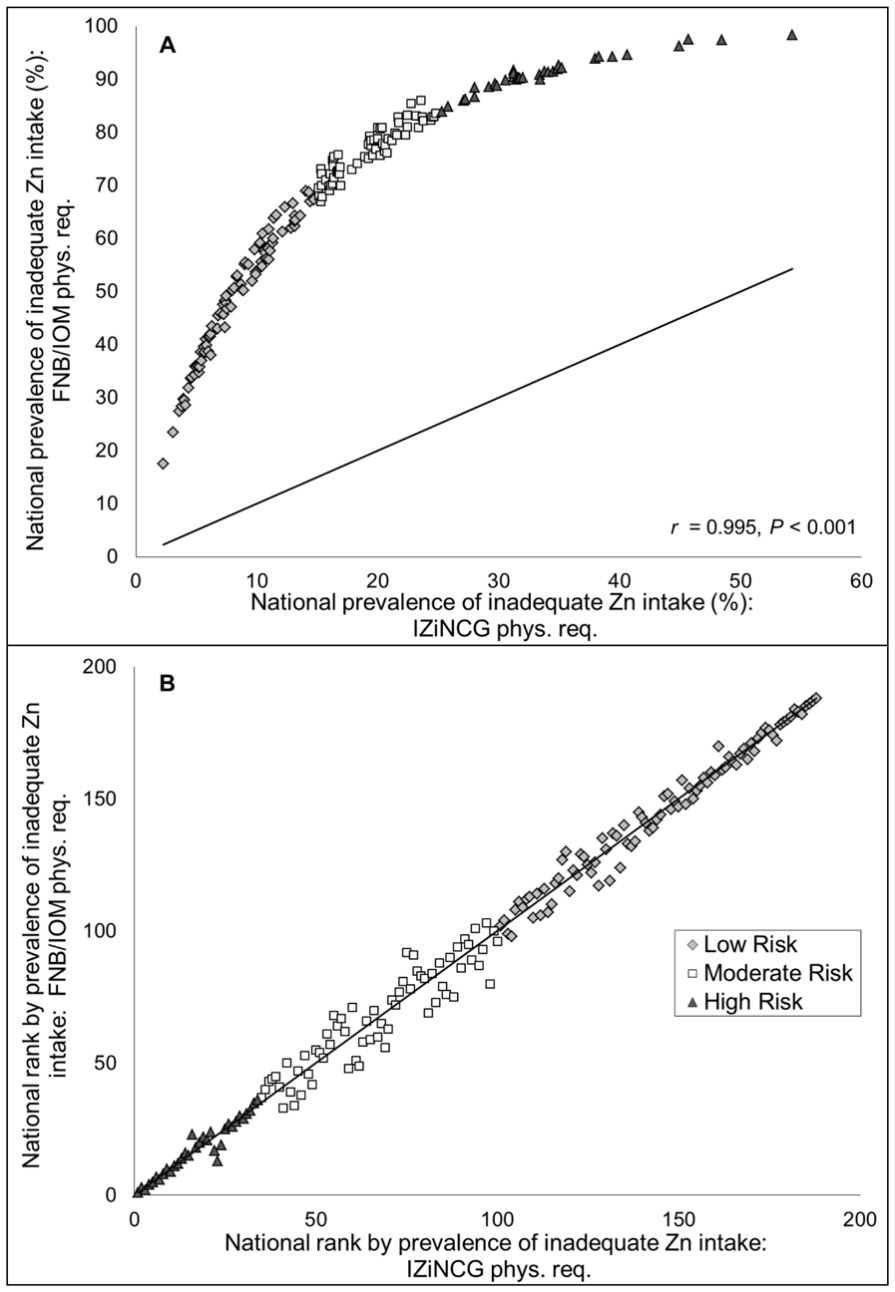
Associations between the (A) national prevalence and (B) country-specific rank order by prevalence of inadequate zinc intake as estimated by different physiological requirements. Estimates are based on the physiological requirements developed by the International Zinc Nutrition Consultative Group (IZiNCG) and the Food and Nutrition Board of the Institute of Medicine (FNB/IOM). The composite nutrient database, the Miller equation for absorbed zinc and a 25% inter-individual CV in intake are used for all estimates. The solid line represents the line of identity (intercept = 0, slope = 1). N = 188 countries. Country rank orders were assigned in descending order, with the country with the highest estimated prevalence of inadequate zinc intake having the lowest rank.

Holding assumptions regarding the nutrient composition database (composite) and absorbed zinc (Miller equation) constant, FNB/IOM physiological requirements estimate a global prevalence of inadequate intake of 66.1%, compared to the 17.3% estimated when applying the IZiNCG physiological requirements (**Table 3**). With the FNB/IOM physiological requirements, the regional prevalence of inadequate intake ranged from 45.2% in high-income regions to 88.6% in South Asia; the lowest country-specific prevalence of inadequate intake was 17.4% (Argentina), and the highest estimated prevalence was 98.8% (Zimbabwe) **(** **Figure 2a**). The dramatically elevated prevalence of inadequate zinc intake with the FNB/IOM physiological requirements results from a global mean physiological requirement, calculated using FNB/IOM recommendations, which was 50% greater than that calculated using IZiNCG recommendations (3.0 vs. 2.0 mg/capita/d, respectively). The global mean estimated availability of absorbable zinc in national food supplies was 2.7 mg/capita/d. Although the estimated prevalence of inadequate zinc intake changed dramatically when different physiological requirements were applied, country-specific rank order of estimated prevalence of inadequate zinc intake was highly conserved (*r* = 0.995, *P*<0.001) **(** **Figure 2b**).

### Effect of Different Estimated Dietary Requirements on the Estimated Prevalence of Inadequate Zinc Intake

**Figure 3:**
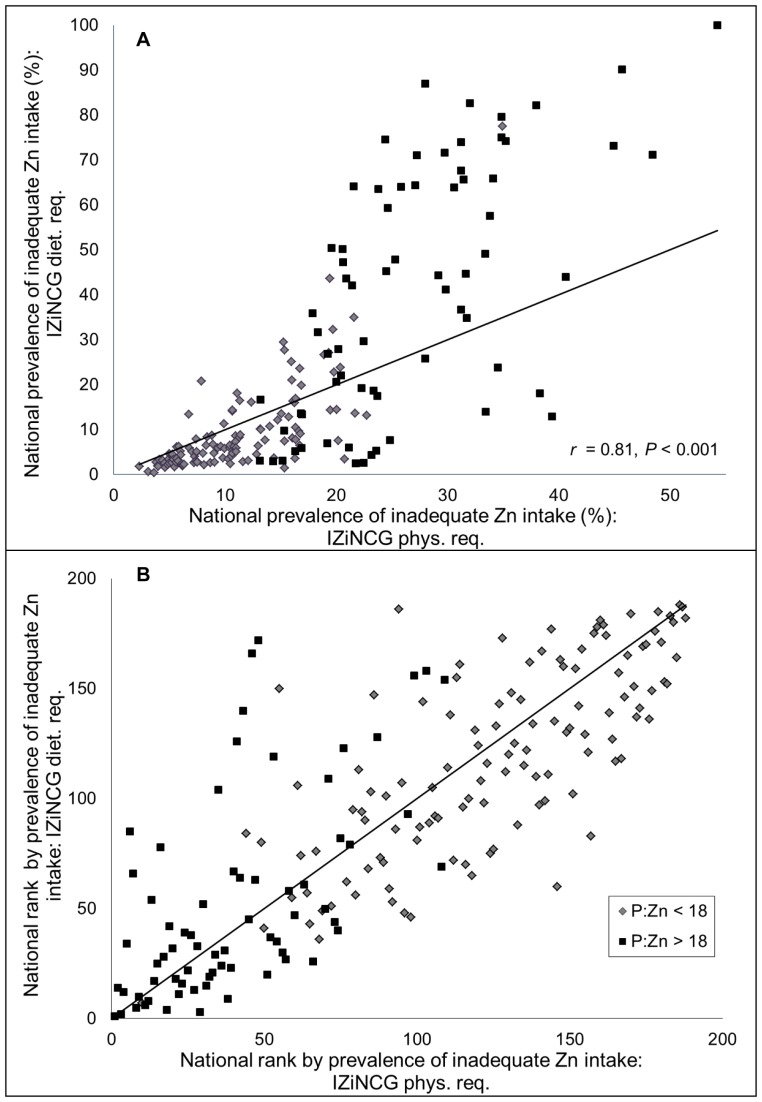
Associations between the (A) national prevalence and (B) country-specific rank order by prevalence of inadequate zinc intake as estimated by different IZiNCG Zn requirements. Estimates are based on physiological and dietary zinc requirements developed by the International Zinc Nutrition Consultative Group. The composite nutrient database and a 25% inter-individual CV in intake are used for all estimates. The solid line represents the line of identity (intercept = 0, slope = 1). N = 188 countries. Country rank orders were assigned in descending order, with the country with the highest estimated prevalence of inadequate zinc intake having the lowest rank.

IZiNCG’s proposed estimated average dietary zinc requirements, developed for the purpose of international applications, rather than for North American populations, are specific to life stage and diet type (P:Zn molar ratio). The global estimate of the prevalence of inadequate zinc intake based upon IZiNCG dietary requirements was similar to that generated based upon IZiNCG physiological requirements (20.8% vs. 17.3%, respectively) (**Table 3** **)**. However, there was considerable variability in the two sets of results for individual countries **(** **Figure 3a** **)**. Among countries with a P:Zn molar ratio≤18, the mean estimates were similar, although the range of estimates was much greater when IZiNCG dietary requirements were applied than when the physiological requirements were used (9.4% (0.34–77.5%) vs. 11.1% (2.3–34.9), respectively). However, among countries with a P:Zn molar ratio >18, both the mean and range of estimates of the prevalence of inadequate zinc intake were dissimilar when IZiNCG dietary requirements were applied (IZiNCG dietary requirement: 40.8% (2.5–99.9%) vs. IZiNCG physiological requirement: 26.8% (13.1–54.3)). The prevalence of inadequate zinc intake was greater with IZiNCG dietary requirements than with the physiological requirements in all regions except Central Asia, North Africa and the Middle East, where total zinc availability was high enough to compensate for the higher dietary requirements. IZiNCG dietary requirements are based upon IZiNCG physiological requirements and were calculated based on a FAZ of ∼30% and ∼22% (a difference of 8%) when the P:Zn molar ratios are≤18 and >18, respectively. By contrast, with the Miller equation, the difference in estimated mean FAZ between countries with a P:Zn molar ratio≤18 and >18 was only 2.2% (25.2% and 23.0%, respectively). However, the Miller Equation captured considerable inter-country variability in the FAZ (range, 13.4–37.8%), which was not accounted for in the establishment of the IZiNCG dietary requirements. Nevertheless, country-specific rank order of estimated prevalence of inadequate zinc intake was conserved between estimates generated using IZiNCG physiological and dietary requirements (r = 0.81, P<0.001) (**Figure 3b**).

**Figure 4:**
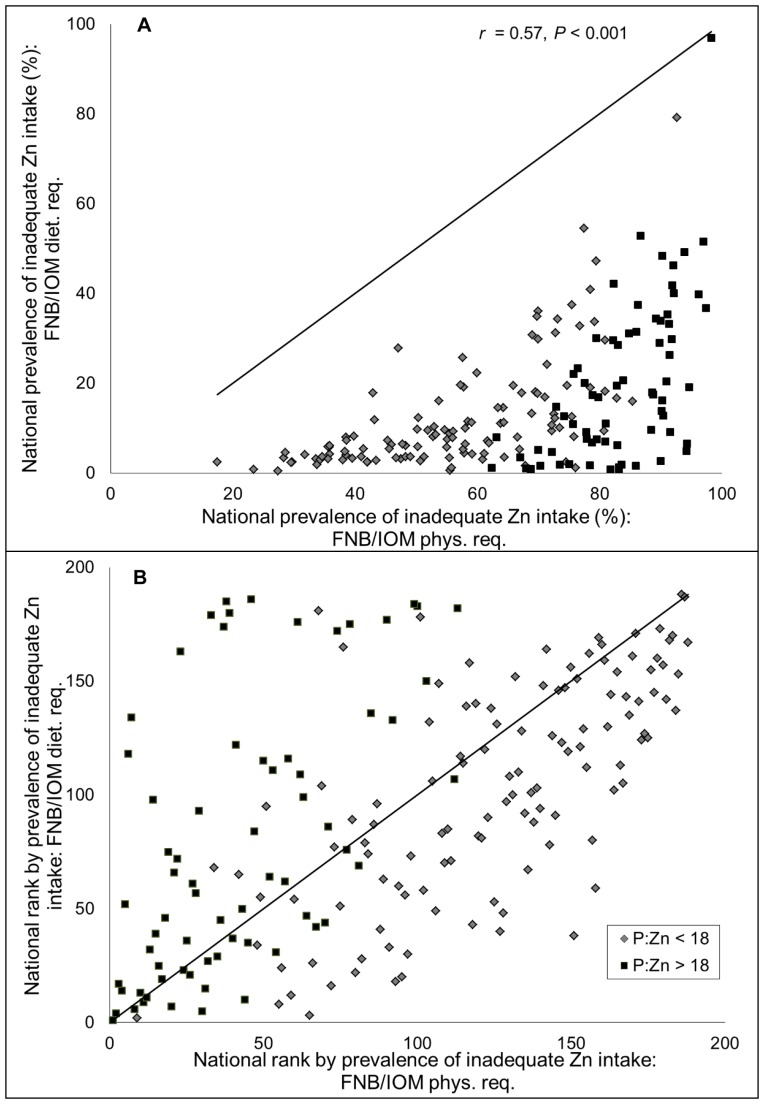
Associations between the (A) national prevalence and (B) country-specific rank order by prevalence of inadequate zinc intake as estimated by different FNB/IOM Zn requirements. Estimates are based on physiological and dietary zinc requirements developed by the Food and Nutrition Board of the Institute of Medicine. The composite nutrient database and a 25% inter-individual CV in intake are used for all estimates. The solid line represents the line of identity (intercept = 0, slope = 1). N = 188 countries. Country rank orders were assigned in descending order, with the country with the highest estimated prevalence of inadequate zinc intake having the lowest rank.

All country- and region-specific, as well as the global, estimated prevalence of inadequate zinc intake based upon FNB/IOM physiological requirements were dramatically higher than those based upon FNB/IOM dietary requirements (global mean prevalence 66.1% vs. 12.1%, respectively) (**Table 3** **;** **Figure 4a** **)**. The fractional absorption of zinc as estimated by the Miller equation and used to calculate the estimated prevalence of inadequate intake in relation to physiological requirements was 24.3±3.6% (global, weighted by national population size; range = 13.4–35.6%); however, FNB/IOM dietary requirements are based on a fixed fractional absorption of zinc of 41%, irrespective of dietary intake. Due to the universal application of a single FAZ estimate, country-specific rank order improved in countries with higher P:Zn molar ratios relative to those with lower P:Zn molar ratios; overall country-specific rank order was only moderately conserved (r = 0.57, *P*<0.001) (**Figure 4b** **)**.

### Effect of Different Estimated Inter-individual Coefficient of Variation of Zinc Intake on the Estimated Prevalence of Inadequate Zinc Intake

Holding all other model assumptions constant (composite nutrient database, IZiNCG physiological requirements and Miller equation), varying the assumed inter-individual CVs from 20 to 30% resulted in a global estimates of the prevalence of inadequate zinc intake ranging from 13.1 to 21.0%, respectively.

## Discussion

The present analyses suggest a high prevalence of inadequate zinc intake, particularly in the regions of South Asia and Sub-Saharan Africa; however, the absolute prevalence estimates vary depending on the assumptions applied with regard to nutrient composition of commodity foods, estimated zinc requirements, and inter-individual variability in zinc intake.

However, all models suggest that inadequate zinc intake may be fairly common. With one exception, the new estimates of the global prevalence of inadequate zinc intake ranged from 12–21% among models, and are generally consistent with our previous estimate of 20.5% [8]. The exception was the estimate (66%) obtained by applying FNB/IOM physiological requirements, which seem to be an overestimate of true requirements, because even relatively affluent countries in Europe and North America had elevated prevalence of inadequate intakes using this model.

Because of the multiple sources of uncertainty in in these food balance sheet models which estimate the prevalence of inadequate zinc intake, considerable caution needs to be used in the interpretation of the prevalence figures, and they should not be considered as numeric absolutes. However, when countries are ranked according to the estimated prevalence of inadequate intake in their population, the country-specific rank order is fairly consistent across assumptions, except for the comparison between FNB/IOM physiological and dietary requirements. Thus, by using the information on rank order, inter-country inferences can be drawn regarding the relative likelihood of zinc deficiency as a public health problem. Countries at high-risk can be targeted for further assessments of population zinc status using measurements of plasma zinc concentration and dietary zinc intake as part of nationally representative nutritional assessment surveys.

The estimate based on FAO FBS is advantageous in the preliminary identification of countries deemed to be at high risk of zinc deficiency relative to other countries, insofar as it uses relevant national food balance data that are routinely collected, standardized and presently available in the public domain. However, there are several potential limitations of this method that need to be recognized in the interpretation and subsequent use of the results, as described in the following paragraphs.

### FAO Food Balance Sheet Data

Attempts were made in the present analysis to identify outliers and to impute values when data were missing or considered implausible; however, inaccuracies may continue to exist in the national food balance data as reported to the FAO. Moreover, the type and extent of processing applied to food commodities are not reported in the FAO food balance sheets. However, in previous analyses of the impact of applying different assumptions regarding the processing of wheat and maize, these differences had relatively little effect on the estimated percentage of the mean physiological requirement available in the national food supply (8). In addition, food balance sheets supply data on annual food availability, and the present analysis does not account for, inter- and intra-household differences in the distribution of food to individuals, or food wastage within the household, all of which may substantially affect dietary zinc intake, or seasonal variation in food supply which may differentially affect zinc status throughout the year [33].

### Zinc and Phytate Contents of Food Commodities

When the composite, IML or NDSR nutrient composition databases were applied to the model, holding all other assumptions constant, the global prevalence of inadequate zinc intake ranged from 13–21%. The largest differences in estimates occurred in Asian regions, largely due to the different values for zinc, phytate and resulting P:Zn molar ratios, of rice in the three databases (P:Zn molar ratios: Composite = 20.4; IML = 31.2; NDSR = 10.3). Overall, we found large variations in the reported zinc and phytate contents of individual foods among different regional and universal food composition databases, and within the scientific literature. Inter-database variation may be due to actual differences in the zinc and phytate contents of similar foods, possibly related to differences in genetics or environmental conditions [34]–[38]. However, the type and extent of processing applied to the analyzed foods and different methods of laboratory analysis, may be an important contributor to inter-database variation reported zinc and phytate contents. National, regional and universal food composition databases need to be further developed, with an emphasis on inclusion of phytate data, source documentation, and periodic updating and revision of estimates.

Additional information is needed on the prevalence of country- and region-specific food processing methods, and the impact various practices have on the total zinc and phytate contents of the foods and zinc bioavailability. In the present analyses, we focused on decortication, milling, fermentation and nixtamalization of cereals, and fermentation of starchy roots. However, we recognize that there is a serious lack of published information, and that inter-individual, national and regional differences in the type, order and duration of processing methods can dramatically influence the resultant zinc and phytate contents of the food commodities. For example, research has shown that extent of decortication of millet in Sahelian West Africa are highly dependent on the woman operator of the mortar and pestle [37]. The zinc and phytate content of raw rice samples obtained from local markets in Sri Lanka was found to differ 10-fold, due in part to the degree of milling, polishing and parboiling [39]. In Bangladesh, 16% of the Zn present in milled rice was lost upon cooking when the excess cooking water was discarded [40].

From 2003–2007, very few countries had implemented mandatory national programs for the zinc fortification of wheat and/or maize flour [19]; therefore, the present analyses do not take into account the impact of zinc fortification programs on the estimated prevalence of inadequate zinc intake. However, since the publication of the WHO and FAO document “Guidelines on food fortification with micronutrients” in 2006 [41], additional countries have implemented or are planning national zinc fortification programs. As of 2012, approximately 20–25 countries have enacted laws regarding mandatory fortification programs (personal communication, Flour Fortification Initiative). As mandatory national zinc fortification programs become more prevalent, and as FAO FBS data become available for the relevant fortification years, these analyses should be repeated and used to simulate changes in the estimated prevalence of inadequate zinc intake with and without fortification.

### Theoretical Zinc Requirements

The WHO/FAO/IAEA, FNB/IOM, and IZiNCG have each proposed different physiological requirements for absorbed zinc, as well as dietary requirements for total zinc intake [1], [22], [27], [42]. The WHO physiological requirements for absorbed zinc are based on a limited number of predominantly single-meal studies in which zinc intake was severely restricted; therefore, resulting estimates may not reflect true physiological requirements when zinc intake is marginal or sufficient [42]. Given these concerns, the WHO estimated requirements were not used in the present analysis, which focused instead on both physiological and dietary zinc requirements developed by the FNB/IOM and IZiNCG. Although the FNB/IOM and IZiNCG followed the same conceptual approach to estimate physiological zinc requirements, those estimated by the FNB/IOM are markedly higher than those estimated by IZiNCG. This discrepancy is due to the inclusion of additional data from studies involving a wider spectrum of age, sex and geographical region in the latter analysis, as well as the use of different reference body weights and statistical weighting methods [43], [44]. It should be noted the factorial approach used to estimate physiological requirements relies upon linear regression to estimate zinc absorption from zinc intake, and both sets of estimated requirements based on the regression analysis are within each other’s confidence interval, thus emphasizing the lack of precision in these existing estimates [1]. However, the higher physiological zinc requirements of the FNB/IOM result in seemingly unrealistic estimates of the global prevalence of inadequate zinc intake, even in high-income countries, where the estimated prevalence approached 50%. Estimates of physiological zinc requirements should be re-evaluated to reconcile differences, using both existing data and new information from individuals with a broader range of ages and zinc intakes, as new studies become available. Revised estimates of physiological zinc requirements may have a dramatic impact on the estimates of the prevalence of inadequate zinc intake, using the food balance sheet approach or dietary intake surveys.

All analyses using physiological requirements applied the Miller equation, a physiologically based mathematical model using saturation kinetics that considers the effects of zinc and phytate intakes on Zn absorption [20]. This newer model differs from the previous analyses conducted by this group [8], which relied upon the purely mathematical logit model developed by IZiNCG [1]. Although the Miller equation models data from ∼70 studies of zinc absorption, data are lacking for young children and populations with high phytate intakes, so current estimates of absorbed zinc may need to be modified as new information on zinc absorption becomes available. In addition, the accuracy of estimates of absorbed zinc using the Miller equation depends on the validity of the data on phytate content of the food commodities.

The FNB/IOM dietary requirements likely underestimate the prevalence of inadequate zinc intake, due to an assumed fractional absorption of zinc (41%) that is much higher than that predicted by the Miller equation in this analysis (24%). FNB/IOM dietary requirements are based on zinc absorption data from low-phytate or phytate-free diets, and thus their application does not seem justifiable for the global estimation of the prevalence of inadequate zinc intake. IZiNCG proposed higher dietary requirements for total zinc intake among individuals consuming diets with low zinc bioavailability (P:Zn molar ratios >18), in a preliminary attempt to address the impact of phytate intake on zinc absorption. In the present analysis, the estimated prevalence of inadequate zinc intake in the global population was similar when IZiNCG physiological or dietary requirements were applied and country-specific rank order was well conserved. However, the IZiNCG dietary requirements are based on limited data and the dichotomous categorization by diet type. This simplification, compared to results obtained when the Miller equation is used to calculate FAZ based on country-specific zinc and phytate dietary intakes, results in a wider range of estimates of the prevalence of inadequate zinc intake, and potentially overestimates dietary zinc inadequacy in those consuming diets with low zinc bioavailability.

### EAR Cut-point Method

Our estimate of the prevalence of inadequate zinc intake is based on a method akin to the EAR cut-point method, the theoretical aspects of which have been previously described [26]. The present analyses assume a 25% CV in inter-individual differences in intake, based on a limited number of existing datasets. As country-specific data become available on the true inter-individual CV across age and sex groups in respective populations, the present estimates should be updated. However, applying assumptions of 20% and 30% inter-individual CVs in intake altered the estimated prevalence of inadequate zinc intake by less than ±5%, and did not affect the country-specific rank order, suggesting that the refinements will not have a large impact on the estimated global prevalence of inadequate zinc intake.

The following information is necessary strengthen the present analysis: (1) further development of national and regional food composition tables, with the inclusion of phytate data; (2) information on local food processing methods, focusing on major sources of zinc and phytate; (3) additional modeling of data on zinc absorption among different populations and population subgroups of varying age and dietary practices; (4) consensus on the estimation of physiological and estimated average dietary requirements, accounting for phytate intake; and (5) information from nationally representative dietary surveys on inter-individual variability in intakes. As this information becomes available, the estimates put forth in this analysis can be easily updated and modified. In addition, national estimates of the prevalence of inadequate zinc intake obtained from analyses of the food balance sheets should be validated against direct indicators of a population’s risk of zinc deficiency, including nationally representative dietary data and plasma zinc concentrations, as this information becomes available.

These analyses indicate that the prevalence of inadequate zinc intake may be fairly common. However, there is considerable variability in the estimates based on the application of different model assumptions, most strikingly between different theoretical mean physiological requirements for zinc. Given the current state of knowledge, we generated a model considered to provide the best estimate of the prevalence of inadequate zinc intake. This “best-estimate” model, comprised of zinc and phytate data from the composite nutrient database, IZiNCG physiological requirements for absorbed zinc, the Miller equation to estimate total zinc absorption, and a 25% CV in inter-individual variation in intakes, indicates that the zinc content of national food supplies may be inadequate to meet zinc requirements for ∼17% of the world’s population. This model owes it strength to the thoroughness of this analysis’ review of food composition databases, regional food processing techniques, and zinc requirements and absorption. However, based on the current state of knowledge, we do not believe that this approach using FAO food balance sheets can be used to estimate the true prevalence of inadequate zinc intake. Nevertheless, because country-specific rank order was highly conserved across most estimates, information obtained from these analyses can be used to draw inferences regarding the relative likelihood of zinc deficiency as a public health problem in different countries. These results suggest that the prevalence of zinc deficiency is greatest in South Asia and Sub-Saharan Africa; further assessment of zinc status, using plasma zinc concentration and/or dietary zinc intake as part of nationally representative nutritional assessment surveys should be a priority in these regions.

## Supporting Information

Table S1 **Nutrient composition data for standardized food commodities, as defined by the Food and Agriculture Organization of the United Nations and obtained from different nutrient composition databases.** Individual foods were aggregated into standardized food commodities and weighting factors were applied based on the number of foods defined for each commodity. The zinc and phytate contents of the individual foods are provided, as appropriate, for the USDA Nutrient Database for Standard Reference, Release 23 (USDA SR23), Nutrition Data System for Research (NDSR), WorldFood System International Mini-list (IML) and composite database created for the purposes of this analysis.
(XLS)

Table S2 **Regional extraction and processing assumptions for cereals, starchy roots and legumes, and the effects of extraction and processing on the zinc and phytate contents of the standardized food commodities.**
(XLS)

Table S3 **Documentation of sources used in the regional extraction and processing assumptions for cereals, starchy roots and legumes, and the effects of extraction and processing on the zinc and phytate contents of the standardized food commodities.**
(DOCX)

Table S4 **Regional classifications.**
(DOCX)
