# Supplementary material for: Estimating the Global Prevalence of Inadequate Zinc Intake from National Food Balance Sheets: Effects of Methodological Assumptions
Source: PLoS One. 2012 Nov 29;7(11):e50565. doi: 10.1371/journal.pone.0050565 (PMC3510064; doi:10.1371/journal.pone.0050565)
Supplement: Table S3 — Documentation of sources used in the regional extraction and processing assumptions for cereals, starchy roots and legumes, and the effects of extraction and processing on the zinc and phytate contents of the standardized food commodities. (DOCX) [file pone.0050565.s003.docx]

**Wheat:**

1. Percent and rate of extraction:
2. Wuehler SE, Peerson JM, Brown KH (2005) Use of National Food Balance Data to Estimate the Adequacy of Zinc in National Food Supplies: Methodology and Regional Estimates. Public Health Nutr 8:812-9.
3. Begin F, Greig A (2002) Food Fortification in West Africa: Assessment of Opportunities and Strategies. The Micronutrient Initiative. pp. 21.
4. Faridi H, Faubion JM, editors (1995) Wheat End Uses Around the World. St. Paul, MN: American Association of Cereal Chemists.
5. Johnson Q. Coordinator, Training and Technical Support Group, The Flour Fortification Initiative (personal communication, March 2011).
6. Prakash V. Nutrition Society of India (personal communication, May 2011)
7. Effect of extraction on Zn and Phytate values:
8. The ratios of remaining nutrients in extracted flour (Zn and phytate mg/100g) were calculated by dividing the values of "ingredient, flour, white all-purpose, unenriched"(NDSR 29484) by "ingredient, flour, whole wheat, use in recipes not containing yeast" (NDSR 1822) using the Nutrition Data System for Research software Version 2010, developed by the Nutrition Coordinating Center (NCC), University of Minnesota, Minneapolis, MN. Schakel S (2001) Maintaining a nutrient database in a changing marketplace: Keeping pace with changing food products - A research perspective. J Food Comp Anal 14: 315-322.
9. Percent fermented:
10. Wuehler SE, Peerson JM, Brown KH (2005) Use of National Food Balance Data to Estimate the Adequacy of Zinc in National Food Supplies: Methodology and Regional Estimates. Public Health Nutr 8:812-9.
11. Faridi H, Faubion JM, editors (1995) Wheat End Uses Around the World. St. Paul, MN: American Association of Cereal Chemists.
12. Johnson Q. Coordinator, Training and Technical Support Group, The Flour Fortification Initiative (personal communication, March 2011).
13. He Z, Xia X, Zhang Y (2010) Breeding Noodle Wheat in China. In: Hou G, editor. Asian Noodles: Science, Technology and Processing. Hoboken, NJ: John Wiley and Sons, Inc.
14. Effect of fermentation on phytate values:
15. Calloway D, Murphy S, Bunch S (1994) User’s guide to the International Mini-list Nutrient Database (a component of the WorldFood Dietary Assessment System). Developed under USAID Cooperative Agreement No. N 5116-A-00-2030-00. Department of Nutritional Sciences, University of California at Berkeley, Berkeley, CA.

**Maize:**

1. Percent and rate of extraction:
2. Mejia D, editor (2003) Maize: Post-Harvest Operation. Food and Agriculture Organization of the United Nations. Available: <http://www.fao.org/fileadmin/user_upload/inpho/docs/Post_Harvest_Compendium_-_MAIZE.pdf>. Accessed 21 April 2011.
3. Food and Agriculture Organization of the United Nations (1992) Maize in Human Nutrition. Rome, Italy: Food and Agriculture Organization of the United Nations. Available: <http://www.fao.org/docrep/T0395E/T0395E00.htm>. Accessed 21 April 2011.
4. Nuss ET, Tanumihardjo SA (2010) Maize: A Paramount Staple Crop in the Context of Global Nutrition. Comprehensive Reviews in Food Science and Food Safety 9: 417-436.
5. To Feed Ourselves (1985) A Proceedings of the First Eastern, Central and Southern Africa Regional Maize Workshop. Lusaka, Zambia.
6. Johnson Q. Coordinator, Training and Technical Support Group, The Flour Fortification Initiative (personal communication, March 2011).
7. Iron Working Group (2008) Background Document II. Second Technical Workshop on Wheat Flour Fortification. Atlanta, GA, USA. Available: <http://www.sph.emory.edu/wheatflour/atlanta08/Atlantabackgrounddocument2finaldraft.pdf>. Accessed 18 March 2011.
8. Guantai S, Seward P (2010) Maize Milling. In: Guantai S, Seward P, editors. Kenya Maize Handbook. Nairobi, Kenya: ACDI/VOCA-Kenya, Kenya Maize Development Programme. pp. 159-170.
9. Razafindrazaka,V. World Food Program (personal communication, March 2011)
10. Hotz, C. Nutridemics (personal communication, March 2011).
11. Mouquet-Rivier, C. Institut de Recherche pour le developpement (personal communication, May 2011).
12. Nago M, Hounhouigan J, Akissoe N, Zanou E, Mestres C (1998) Characterization of the Beninese traditional ogi, a fermented maize slurry: physicochemical and microbiological aspects. International Journal of Food Science and Technology 33: 307-315.
13. Janer Z (2008) Latino Food Culture. Westport, Connecticut: Greenwood Press. 200 p.
14. LeClerq, S. Johns Hopkins Center for Global Health (personal communication March 2011)
15. Effect of extraction on zinc and phytate values
16. The ratio of remaining nutrients in extracted flour (Zn mg/100g) was calculated by dividing the values of "ingredient, cornmeal - dry, white (degermed, enriched)" (NDSR 28899) by "ingredient, flour, corn, whole grain, white" (NDSR 111508) using the Nutrition Data System for Research software Version 2010 developed by the Nutrition Coordinating Center (NCC), University of Minnesota, Minneapolis, MN. Schakel S (2001) Maintaining a nutrient database in a changing marketplace: Keeping pace with changing food products - A research perspective. J Food Comp Anal 14: 315-322. Note: NDSR nutrient profiles for 28899 and 111508 do not include a concomitant reduction in phytate as well as zinc, thus other sources were used.
17. US Department of Agriculture (USDA) USDA Nutrient Database for Standard Reference, Release 23 [online]. Available: <http://www.nal.usda.gov/fnic/cgi-bin/nut_search.pl>. Accessed: 16 August 2011. (Remaining Zn = 0.36).
18. Iron Working Group (2008) Background Document II. Second Technical Workshop on Wheat Flour Fortification. Atlanta, GA, USA. Available: <http://www.sph.emory.edu/wheatflour/atlanta08/Atlantabackgrounddocument2finaldraft.pdf>. Accessed 18 March 2011.
19. Ferguson EL, Gibson RS, Thompson LU, Ounpuu S, Berry M (1988) Phytate, zinc, and calcium contents of 30 East African foods and their calculated phytate:Zn, Ca:phytate, and [Ca][phytate]/[Zn] molar ratios. J Food Comp Anal 1: 316-325.
20. Percent fermented:
21. Mejia D, editor (2003) Maize: Post-Harvest Operation. Food and Agriculture Organization of the United Nations. Available: <http://www.fao.org/fileadmin/user_upload/inpho/docs/Post_Harvest_Compendium_-_MAIZE.pdf>. Accessed 21 April 2011.
22. Food and Agriculture Organization of the United Nations (1992) Maize in Human Nutrition. Rome, Italy: Food and Agriculture Organization of the United Nations. Available: <http://www.fao.org/docrep/T0395E/T0395E00.htm>. Accessed 21 April 2011.
23. Wuehler SE, Peerson JM, Brown KH (2005) Use of National Food Balance Data to Estimate the Adequacy of Zinc in National Food Supplies: Methodology and Regional Estimates. Public Health Nutr 8:812-9.
24. Johnson, Q. Coordinator, Training and Technical Support Group, The Flour Fortification Initiative (email communication, March 2011).
25. Good S (2009) Animal source foods and nutrition during early life: an evaluation of the possible link between livestock keeping, food intake and nutritional status of young children in Ethiopia [Dissertation]. Swiss Federal Institute of Technology Zürich, Switzerland. Diss ETH No. 18483. doi:10.3929/ethz-a-005940277. 185 p.
26. Umeta M, West CE, Fufa H (2005) Content of zinc, iron, calcium and their absorption inhibitors in foods commonly consumed in Ethiopia. J Food Comp Anal 18: 803-817. (wheat/ teff data)
27. Abebe Y, Bogale A, Hambidge KM, Stoecker BJ, Bailey K, et al. (2007) Phytate, zinc, iron and calcium content of selected raw and prepared foods consumed in rural Sidama, Southern Ethiopia, and implications for bioavailability. J Food Comp Anal 20: 161-168. (wheat/ teff data)
28. Nago M, Hounhouigan J, Akissoe N, Zanou E, Mestres C (1998) Characterization of the Beninese traditional ogi, a fermented maize slurry: physicochemical and microbiological aspects. International Journal of Food Science and Technology 33: 307-315.
29. Moquet-Rivier, C. Institut de Recherche pour le developpement (personal communication May 2011)
30. Staubli Asobayire F (2000) Development of a food fortification strategy to combat iron deficiency in the Ivory Coast [Dissertation]. Swiss Federal Institute of Technology Zürich, Switzerland. Diss ETH No. 13730. doi:10.3929/ethz-a-004041447. 74 p.
31. Effect of fermentation on phytate values:
32. Reddy N, Sathe S (2002) Food Phytates. Boca Raton, FL: CRC Press LLC. 280 p.
33. Good S (2009) Animal source foods and nutrition during early life: an evaluation of the possible link between livestock keeping, food intake and nutritional status of young children in Ethiopia [Dissertation]. Swiss Federal Institute of Technology Zürich, Switzerland. Diss ETH No. 18483. doi:10.3929/ethz-a-005940277. 185 p. (wheat/ teff data)
34. Umeta M, West CE, Fufa H (2005) Content of zinc, iron, calcium and their absorption inhibitors in foods commonly consumed in Ethiopia. J Food Comp Anal 18: 803-817. (wheat/ teff data)
35. Abebe Y, Bogale A, Hambidge KM, Stoecker BJ, Bailey K, et al. (2007) Phytate, zinc, iron and calcium content of selected raw and prepared foods consumed in rural Sidama, Southern Ethiopia, and implications for bioavailability. J Food Comp Anal 20: 161-168. (wheat/ teff data)
36. Marfo EK, Simpson BK, Idowu JS, Oke OL (1990) Effect of local food processing on phytate levels in cassava, cocoyam, yam, maize, sorghum, rice, cowpea, and soybean. J Agric Food Chem 38: 1580-1585.
37. Ejigui J, Savoie L, Marin J, Desrosiers T (2005) Beneficial changes and drawbacks of a traditional fermentation process on chemical composition and antinutritional factors of yellow maize (*Zea mays*). Journal of Biological Sciences 5: 590-596.
38. Proulx A, Reddy M (2007) Fermentation and Lactic Acid Addition Enhance Iron Bioavailability of Maize. J Agric Food Chem 55: 2749-2754.
39. Iron Working Group (2008) Background Document II. Second Technical Workshop on Wheat Flour Fortification. Atlanta, GA, USA. Available: <http://www.sph.emory.edu/wheatflour/atlanta08/Atlantabackgrounddocument2finaldraft.pdf>. Accessed 18 March 2011.
40. Bressani R, Turcios JC, Colmenares de Ruiz AS, de Palomo PP (2004) Effect of processing conditions on phytic acid, calcium, iron, and zinc contents of lime-cooked maize. J Agric Food Chem 52: 1157-1162.

**Millet and Sorghum:**

1. Percent and rate of extraction
2. Food and Agriculture Organization of the United Nations (1995) Sorghum and Millets in Human Nutrition. Rome, Italy: Food and Agriculture Organization of the United Nation. Available: <http://www.fao.org/docrep/T0818E/T0818E01.htm>. Accessed 24 March 2011.
3. Kajuna S, editor. Chapter XVIII Millet: Post-Harvest Operations: Food and Agriculture Organization of the United Nations. Available: <http://www.fao.org/inpho/content/compend/text/ch18.htm>. Accessed 24 March 2011.
4. Leder I Sorghum and Millets. In: Fuleky G, editor. Cultivated Plants, Primarily as Food Sources. UNESCO - Encyclopedia of Life Support Systems.
5. Vogel S, Graham M (1979) Sorghum and Millet: Food Production and Use. Report of a workshop held in Nairobi, Kenya, 4-7 July 1978. Ottawa, Canada: International Development Research Centre.
6. Anglani C (1998) Sorghum for Human Food - A Review. Plant Foods for Human Nutrition 52: 85-95.
7. Taylor J Overview: Importance of Sorghum in Africa. Department of Food Science, University of Pretoria, Pretoria 0002, South Africa (E-mail: [jtaylor@postino.up.ac.za](mailto:jtaylor@postino.up.ac.za)).
8. Mouquet-Rivier C. Institut de Recherche pour le developpement (personal communication May 2011).
9. Hash, CT. Principal Scientist, ICRISAT-Sadore, Niger (personal communication May 2011)
10. Hama F, Icard-Verniere C, Guyot JP, Picq C, Diawara B, et al. (2011) Changes in micro- and macronutrient composition of pearl millet and white sorghum during in field versus laboratory decortication. Journal of Cereal Science 54: 425-433.
11. Buerkert A, Moser M, Kumar AK, Fuerst P, Becker K (2001) Variation in grain quality of pearl millet from Sahelian West Africa. Field Crops Research 69: 1-11
12. LeClerq S. Johns Hopkins Center for Global Health (personal communication March 2011).
13. Waniska R, Rooney L (2002) Sorghum grain quality for increased utilization. In: Leslie J, editor. Sorghum and millet diseases. Ames, IA: Iowa State Press. pp. 327-336.
14. Subramanian V, Jambunathan R, Sambasiva-Rao N (1983) Textural properties of sorghum dough. Journal of Food Science 48: 1650-1673.
15. Prakash V. Nutrition Society of India (personal communication May 2011)
16. Effect of extraction on zinc and phytate values:
17. Calloway D, Murphy S, Bunch S. User’s guide to the International Mini-list Nutrient Database (a component of the WorldFood Dietary Assessment System). Developed under USAID Cooperative Agreement No. N 5116-A-00-2030-00. Department of Nutritional Sciences, University of California at Berkeley, Berkeley, CA, 1994.
18. Reddy N, Sathe S (2002) Food Phytates. Boca Raton, FL: CRC Press LLC. 280 p.
19. Lestienne I, Besancon P, Caporiccio B, Lullien-Pellerin V, Treche S (2005) Iron and zinc in vitro availability in pearl millet flours (Pennisetum glaucum) with varying phytate, tannin, and fiber contents. J Agric Food Chem 53: 3240-3247.
20. Mahgoub S, Elhag S (1998) Effect of milling, soaking, malting, heat treatment and fermentation on phytate level of four Sudanese sorghum cultivars. Food Chemistry 61: 77-80.
21. Percent fermented:
22. Food and Agriculture Organization of the United Nations (1995) Sorghum and Millets in Human Nutrition. Rome, Italy: Food and Agriculture Organization of the United Nations. Available: <http://www.fao.org/docrep/T0818E/T0818E01.htm>. Accessed 24 March 2011.
23. Kajuna S, editor. Chapter XVIII Millet: Post-Harvest Operations: Food and Agriculture Organization of the United Nations. Available: <http://www.fao.org/inpho/content/compend/text/ch18.htm>. Accessed 24 March 2011.
24. Leder I Sorghum and Millets. In: Fuleky G, editor. Cultivated Plants, Primarily as Food Sources. UNESCO - Encyclopedia of Life Support Systems.
25. Vogel S, Graham M (1979) Sorghum and Millet: Food Production and Use. Report of a workshop held in Nairobi, Kenya, 4-7 July 1978. Ottawa, Canada: International Development Research Centre.
26. Anglani C (1998) Sorghum for Human Food - A Review. Plant Foods for Human Nutrition 52: 85-95.
27. Taylor J Overview: Importance of Sorghum in Africa. Department of Food Science, University of Pretoria, Pretoria 0002, South Africa (E-mail: jtaylor@postino.up.ac.za).
28. Good S (2009) Animal source foods and nutrition during early life: an evaluation of the possible link between livestock keeping, food intake and nutritional status of young children in Ethiopia [Dissertation]. Swiss Federal Institute of Technology Zürich, Switzerland. Diss ETH No. 18483. doi:10.3929/ethz-a-005940277. 185 p. (wheat/ teff data).
29. Umeta M, West CE, Fufa H (2005) Content of zinc, iron, calcium and their absorption inhibitors in foods commonly consumed in Ethiopia. J Food Comp Anal 18: 803-817. (wheat/ teff data)
30. Abebe Y, Bogale A, Hambidge KM, Stoecker BJ, Bailey K, et al. (2007) Phytate, zinc, iron and calcium content of selected raw and prepared foods consumed in rural Sidama, Southern Ethiopia, and implications for bioavailability. J Food Comp Anal 20: 161-168. (wheat/ teff data)
31. Effect of fermentation on phytate values:
32. Good S (2009) Animal source foods and nutrition during early life: an evaluation of the possible link between livestock keeping, food intake and nutritional status of young children in Ethiopia [Dissertation]. Swiss Federal Institute of Technology Zürich, Switzerland. Diss ETH No. 18483. doi:10.3929/ethz-a-005940277. 185 p. (wheat/ teff data)
33. Umeta M, West CE, Fufa H (2005) Content of zinc, iron, calcium and their absorption inhibitors in foods commonly consumed in Ethiopia. J Food Comp Anal 18: 803-817. (wheat/ teff data)
34. Abebe Y, Bogale A, Hambidge KM, Stoecker BJ, Bailey K, et al. (2007) Phytate, zinc, iron and calcium content of selected raw and prepared foods consumed in rural Sidama, Southern Ethiopia, and implications for bioavailability. J Food Comp Anal 20: 161-168. (wheat/ teff data)
35. Elyas SHA, Tinay AHE, Yousif NE, Elsheikh EAE (2002) Effect of natural fermentation on nutritive value and in vitro protein digestibility of pearl millet. Food Chemistry 78: 75-79.
36. Reddy N, Sathe S (2002) Food Phytates. Boca Raton, FL: CRC Press LLC. 280 p. (Maize estimates)
37. Eyzaguirre RZ, Nienaltowska K, deJong LE, Hasenack BB, Nout MR (2006) Effect of food processing of pearl millet (Pennisetum glaucum) IKMP-5 on the level of phenolics, phytate, iron and zinc. Journal of the Science of Food and Agriculture 86: 1391-1398.
38. Marfo EK, Simpson BK, Idowu JS, Oke OL (1990) Effect of local food processing on phytate levels in cassava, cocoyam, yam, maize, sorghum, rice, cowpea, and soybean. J Agric Food Chem 38: 1580-1585.
39. Mahgoub S, Elhag S (1998) Effect of milling, soaking, malting, heat treatment and fermentation on phytate level of four Sudanese sorghum cultivars. Food Chemistry 61: 77-80.
40. Proulx A, Reddy M (2007) Fermentation and Lactic Acid Addition Enhance Iron Bioavailability of Maize. J Agric Food Chem 55: 2749-2754.
41. Iron Working Group (2008) Background Document II. Second Technical Workshop on Wheat Flour Fortification. Atlanta, GA, USA. Available: <http://www.sph.emory.edu/wheatflour/atlanta08/Atlantabackgrounddocument2finaldraft.pdf>. Accessed 18 March 2011.
42. Bressani R, Turcios JC, Colmenares de Ruiz AS, de Palomo PP (2004) Effect of processing conditions on phytic acid, calcium, iron, and zinc contents of lime-cooked maize. J Agric Food Chem 52: 1157-1162.

**Cereals, other- teff :**

1. Percent fermented:
2. Good S (2009) Animal source foods and nutrition during early life: an evaluation of the possible link between livestock keeping, food intake and nutritional status of young children in Ethiopia [Dissertation]. Swiss Federal Institute of Technology Zürich, Switzerland. Diss ETH No. 18483. doi:10.3929/ethz-a-005940277. 185 p. (wheat/ teff data)
3. Umeta M, West CE, Fufa H (2005) Content of zinc, iron, calcium and their absorption inhibitors in foods commonly consumed in Ethiopia. J Food Comp Anal 18: 803-817. (wheat/ teff data)
4. Abebe Y, Bogale A, Hambidge KM, Stoecker BJ, Bailey K, et al. (2007) Phytate, zinc, iron and calcium content of selected raw and prepared foods consumed in rural Sidama, Southern Ethiopia, and implications for bioavailability. J Food Comp Anal 20: 161-168. (wheat/ teff data)
5. Effect of fermentation of phytate values:
6. Good S (2009) Animal source foods and nutrition during early life: an evaluation of the

possible link between livestock keeping, food intake and nutritional status of young children in Ethiopia [Dissertation]. Swiss Federal Institute of Technology Zürich, Switzerland. Diss ETH No. 18483. doi:10.3929/ethz-a-005940277. 185 p. (wheat/ teff data)

1. Umeta M, West CE, Fufa H (2005) Content of zinc, iron, calcium and their absorption inhibitors in foods commonly consumed in Ethiopia. J Food Comp Anal 18: 803-817. (wheat/ teff data)
2. Abebe Y, Bogale A, Hambidge KM, Stoecker BJ, Bailey K, et al. (2007) Phytate, zinc, iron and calcium content of selected raw and prepared foods consumed in rural Sidama, Southern Ethiopia, and implications for bioavailability. J Food Comp Anal 20: 161-168. (wheat/ teff data)

**Tubers:**

1. Percent of tubers fermented:
2. Food and Agriculture Organization of the United Nations (1990) Roots, Tubers, Plantains, and Bananas in Human Nutrition. Rome, Italy: Food and Agriculture Organization of the United Nations. Available: <http://www.fao.org/docrep/t0207e/T0207E00.htm>. Accessed: 5 May 2011.
3. Sarma JS (1989) Summary Proceedings of a Workshop on Trends and Prospects of Cassava in the Third World. Washington, D.C.: International Food Policy Research Institute.
4. Food and Agriculture Organization of the United Nations (2000). The World Cassava Economy: Facts, Tends and Outlooks. Available: <http://www.fao.org/docrep/009/x4007e/x4007e00.htm>. Accessed 5 May 2011.

Hahn SK. An overview of traditional processing and utilization of cassava in Africa. In: Hahn SK, Reynolds L, Egbunike GN, editors; 14-18 November 1988; Ibadan, Nigeria. International Institute of Tropical Agriculture.

1. Effect of fermentation on phytate values:
2. Marfo EK, Simpson BK, Idowu JS, Oke OL (1990) Effect of local food processing on phytate levels in cassava, cocoyam, yam, maize, sorghum, rice, cowpea, and soybean. J Agric Food Chem 38: 1580-1585.
3. Food and Agriculture Organization of the United Nations (1990) Roots, Tubers, Plantains, and Bananas in Human Nutrition. Rome, Italy: Food and Agriculture Organization of the United Nations. Available: <http://www.fao.org/docrep/t0207e/T0207E00.htm>. Accessed: 5 May 2011.

**Beans:**

1. Effect of cooking on phytate values:
2. Reddy N, Sathe S (2002) Food Phytates. Boca Raton, FL: CRC Press LLC. 280 p.
