# Supplementary material for: Estimating the Global Prevalence of Inadequate Zinc Intake from National Food Balance Sheets: Effects of Methodological Assumptions
Source: PLoS One. 2012 Nov 29;7(11):e50565. doi: 10.1371/journal.pone.0050565 (PMC3510064; doi:10.1371/journal.pone.0050565)
Supplement: Table S4 — Regional classifications. (DOCX) [file pone.0050565.s004.docx]

| **Region** | **Countries** |
| --- | --- |
|  | |
| High-Income (HIGHIN) | Andorra, Australia, Austria, Belgium, Brunei Darussalam, Canada, Cyprus, Denmark, Finland, France, Germany, Greece, Greenland, Iceland, Ireland, Israel, Italy, Japan, Luxembourg, Malta, Netherlands, New Zealand, Norway, Portugal, Republic of Korea, Singapore, Spain, Sweden, Switzerland, United Kingdom, United States of America |
| Southern and Tropical Latin America (SOTRLA) | Argentina, Brazil, Chile, Paraguay, Uruguay |
| China (CHINAR) | China, Hong Kong SAR (China), Macau SAR (China), Taiwan |
| Central and Eastern Europe (CEEAEU) | Albania, Belarus, Bosnia and Herzegovina, Bulgaria, Croatia, Czech Republic, Estonia, Hungary, Latvia, Lithuania, Macedonia (Former Yugoslav Republic of), Moldova, Montenegro, Poland, Romania, Russian Federation, Serbia, Slovakia, Slovenia, Russian Federation, Ukraine |
| Central and Andean Latin America and the Caribbean (CALACA) | Antigua and Barbuda, Bahamas, Barbados, Belize, Bermuda, Bolivia, British Virgin Islands, Colombia, Costa Rica, Cuba, Dominica, Dominican Republic, Ecuador, El Salvador, Grenada, Guatemala, Guyana, Haiti, Honduras, Jamaica, Mexico, Nicaragua, Netherlands Antilles, Panama, Peru, Puerto Rico, Saint Kitts and Nevis, Saint Lucia, Saint Vincent and the Grenadines, Suriname, Trinidad and Tobago, Venezuela (Bolivarian Republic of) |
| Central Asia, North Africa and Middle East (CANAME) | Algeria, Armenia, Azerbaijan, Bahrain, Egypt, Georgia, Iran (Islamic Republic of), Iraq, Jordan, Kazakhstan, Kuwait, Kyrgyzstan, Lebanon, Libyan Arab Jamahiriya, Mongolia, Morocco, Occupied Palestinian Territory, Oman, Qatar, Saudi Arabia, Syrian Arab Republic, Tajikistan, Tunisia, Turkey, Turkmenistan, United Arab Emirates, Uzbekistan, Yemen |
| East and Southeast Asia and Pacific (ESEASP) | Cambodia, Cook Islands, Democratic People’s Republic of Korea, Fiji, French Polynesia, Indonesia, Kiribati, Lao People's Democratic Republic, Malaysia, Maldives, Marshall Islands, Micronesia (Federated States of), Myanmar, Nauru, Palau, Papua New Guinea, Philippines, Samoa, Solomon Islands, Sri Lanka, Thailand, Timor-Leste, Tonga, Viet Nam, Vanuatu |
| Sub-Saharan Africa (SUSAAF) | Angola, Botswana, Burkina Faso, Burundi, Cameroon, Cape Verde, Central African Republic, Chad, Comoros, Congo, Côte d'Ivoire, Democratic Republic of the Congo, Djibouti, Eritrea, Ethiopia, Equatorial Guinea, Gabon, Gambia, Ghana, Guinea, Guinea-Bissau, Lesotho, Liberia, Kenya, Madagascar, Malawi, Mali, Mauritania, Mauritius, Mozambique, Namibia, Niger, Nigeria, Rwanda, São Tomé and Príncipe, Senegal, Seychelles, Sierra Leone, Somalia, South Africa, Sudan, Swaziland, Togo, Uganda, United Republic of Tanzania, Zambia, Zimbabwe |
| South Asia (SOASIA) | Afghanistan, Bangladesh, Bhutan, India, Nepal, Pakistan |
